# Granulomatous Lymphadenitis as a Manifestation of Q Fever

**DOI:** 10.3201/eid0901.020211

**Published:** 2003-01

**Authors:** Pierre Tattevin, Cédric Arvieux, Mathieu Dupont, Pascal Guggenbuhl, Alexandre Lemeur, Christian Michelet

**Affiliations:** * Hôpital Pontchaillou, Rennes, France; † Hôpital Sud, Rennes, France

**To the Editor:** Q fever is a worldwide zoonosis caused by the obligate intracellular pathogen *Coxiella burnetii* (*1*). Human infection is usually the result of exposure to infected cattle, sheep or goats. Acute Q fever may be asymptomatic or manifest as a self-limiting febrile illness, pneumonia, hepatitis, or meningoencephalitis. Most cases of acute Q fever will resolve without sequelae, but endocarditis, granulomatous hepatitis, osteomyelitis, and endovascular infections are well-documented manifestations of chronic *C. burnetii* infection (*1*). Recently, various atypical manifestations of acute (*2*), and chronic (*3*) Q fever have been reported as well as changing clinical presentation of Q fever endocarditis (*4*) and changing epidemiology of Q fever (*5*). Researchers have suggested that heightened awareness of Q fever among doctors, coupled with improved diagnostic methods, could increase the medical knowledge about this difficult-to-diagnose and difficult-to-treat infection (*4*). We report two cases of granulomatous lymphadenitis associated with *C. burnetii* infection.

A 70-year-old man was admitted to the hospital because of weight loss, night sweats, and a continuous high-grade fever of 2 months’ duration. His past medical history was unremarkable, except for pulmonary tuberculosis treated 55 years earlier and chronic glaucoma. He lived in a rural area and had rare contact with cattle. On admission, his body temperature was 39.5°C; his right laterocervical lymph nodes were enlarged (3 cm x 4 cm) and inflamed. Blood values were unremarkable except for an elevated C-reactive protein level of 150 mg/L (normal<6). A computed tomography scan of the chest showed hilar calcifications and enlarged mediastinal lymph nodes. A biopsy of cervical lymph nodes indicated granulomatous lymphadenitis with foci of necrosis. *C. burnetii* DNA was detected on the lymph nodes with a *C. burnetii*–specific pair of primers that amplified an *htpAB*-associated repetitive element (*6*). Results of serologic testing by indirect immunofluorescence (IF) were positive for *C. burnetii* with immunoglobulin (Ig) G antibody titer to phase 1 and phase 2 antigen of 800 and 1,600, respectively, and IgM antibody titer to phase 2 antigen of 50.

A 44-year-old man was admitted to the hospital because of a continuous low-grade fever of 3 months’ duration. He had worked as a farmer for 15 years and assisted in the birth of sheep and cattle. On admission, his body temperature was 38°C, and right inguinal lymph nodes were inflamed, measuring 4 x 4 cm. A lymph node biopsy showed granulomatous lymphadenitis with stellate abscesses surrounded by palisading epithelioid cells. Serologic testing by indirect IF was positive for *C. burnetii* with an IgG antibody titer to phase 1 antigen of 320.

For both patients, results of Ziehl staining and Lowenstein (Bio-Rad, Marne-La-Coquette, France) cultures of gastric aspirates (x 3) and lymph node specimens were negative for mycobaceria, as were the results of tuberculin skin tests. Other diseases were ruled out, including brucellosis, yersiniosis, bartonellosis, and chlamydial infections (by serologic testing) and fungal infections (parasitologic studies on lymph node tissue). Antinuclear antibodies were absent, and angiotensin-converting-enzyme values were normal. Both patients received doxycycline, 200 mg once a day, and rifampin, 600 mg twice a day, for 1 year, and the symptoms resolved (follow-up at 18 months for patient 1 and 9 months for patient 2, respectively). For patient 1, serologic testing after 1 year of treatment showed an IgG antibody titer to phase 1 antigen of 320.

Granulomatous lymphadenitis has been described during mycobacterial infections, tularemia, cat scratch disease, yersiniosis, lymphogranuloma venereum, histoplasmosis, coccidioidomycosis, and chronic granulomatous diseases (*7*). One well-documented case of acute Q fever with necrotic cervical lymphadenitis has been recently reported (*8*); to our knowledge, granulomatous lymphadenitis has never been reported during Q fever. In both cases reported here, *C. burnetii* was the likely etiologic agent, given the results of polymerase chain reaction and serologic studies (patient 1) or the patient’s occupation and results of the serologic testing (patient 2). Moreover, for both, no other potential cause could be identified, and the response to doxycycline-rifampin regimen was favorable. We suggest that granulomatous lymphadenitis be added to the list of atypical presentations of Q fever.
